# The Accuracy of Simple and Adjusted Aldosterone Indices for Assessing Selectivity and Lateralization of Adrenal Vein Sampling in the Diagnosis of Primary Aldosteronism Subtypes

**DOI:** 10.3389/fendo.2022.801529

**Published:** 2022-02-16

**Authors:** Mirko Parasiliti-Caprino, Fabio Bioletto, Filippo Ceccato, Chiara Lopez, Martina Bollati, Maria Chiara Di Carlo, Giacomo Voltan, Denis Rossato, Giuseppe Giraudo, Carla Scaroni, Ezio Ghigo, Mauro Maccario

**Affiliations:** ^1^ Endocrinology, Diabetes and Metabolism, Department of Medical Sciences, City of Health and Science University Hospital, University of Turin, Turin, Italy; ^2^ Endocrinology Unit, Department of Medicine DIMED, University-Hospital of Padua, Padua, Italy; ^3^ Radiology Unit, City of Health and Science University Hospital, University of Turin, Turin, Italy; ^4^ Surgery, City of Health and Science University Hospital, University of Turin, Turin, Italy

**Keywords:** aldosterone, cortisol, adrenal tumor, hypokalemia, adrenal glands, adrenalectomy, secondary hypertension, endocrine hypertension

## Abstract

**Objective:**

This study aimed to evaluate the reliability of simple and corrected aldosterone indices for assessing the selectivity and lateralization of adrenal vein sampling (AVS) in patients with primary aldosteronism.

**Methods:**

Data of all consecutive patients with primary aldosteronism who underwent AVS for subtype diagnosis, followed at two Italian referral centers, were analyzed retrospectively.

**Results:**

AVS achieved bilateral selectivity in 112/144 patients. Unilateral disease was diagnosed in 60 cases (53.6%) and idiopathic hyperaldosteronism in 52 individuals (46.4%). The aldosterone index (aldosterone ratio between an adrenal vein and the inferior vena cava) showed a high accuracy in predicting selectivity, compared to a cortisol selectivity index of 1.1, and a moderate accuracy, compared to cortisol cut-offs of 2 and 3. The simple aldosterone index showed a moderate accuracy in predicting ipsi/contralateral aldosterone hypersecretion, while lesion side- and hypokalemia-corrected aldosterone index revealed a significant improvement in predicting ipsi/contralateral disease. Moreover, the comparative aldosterone index (aldosterone ratio in the dominant vs the non-dominant adrenal vein) revealed a high accuracy in predicting unilateral primary aldosteronism. For an immediate clinical application of our results, the adjusted cut-offs were calculated, according to the Youden’s criterion and to a pre-established specificity of 90%, for all possible combinations of lesion side at imaging and presence/absence of hypokalemia.

**Conclusions:**

This study demonstrated the diagnostic accuracy of simple and clinical-/imaging-corrected aldosterone indices for adrenal vein sampling in subtype diagnosis of primary aldosteronism and suggests the potential application of these tools to select patients for adrenalectomy when standard indices cannot be performed.

## Introduction

Primary aldosteronism (PA) is a heterogeneous group of disorders caused by an excessive aldosterone secretion that seems autonomous from renin (1–3) and is the most frequent form of secondary hypertension. Its prevalence increases with the severity of hypertension (4, 5), reaching over 29% in individuals with resistant hypertension (6). Diagnosing PA is important to give patients the opportunity of a specific surgical or medical treatment in order to reduce cardiovascular risk (7–9).

The last step of the diagnostic process is the subtype differentiation, in which the adrenal vein sampling (AVS) is currently considered the gold standard, also given the absence of alternative strategies (10). Due to the technical difficulties of its execution, AVS has low diffusion among centers worldwide, with a reported success rate ranging from 26 to 81% (11–14). The reason mainly lies in the difficulty of cannulation of the right adrenal vein, and many studies have demonstrated that an expert radiologist is crucial for improving sampling success (11, 15–17).

The major diagnostic indices for the assessment of lateralization of aldosterone hypersecretion are the lateralization index [LI - dominant vs non dominant adrenal vein aldosterone/cortisol ratio (A/C)], and the contralateral index [CI - non-dominant adrenal vein vs inferior vena cava A/C] (18). These indices, however, can be reliably interpreted only when AVS is bilaterally selective. Therefore, during last years, some authors proposed the use of simple unconventional indices with a moderate (19, 20), and often not reproducible, accuracy for determining PA lateralization (21–24). Most recently, Burrello et al. developed two scores (25, 26) for predicting PA subtypes, using a machine learning approach and reaching a high accuracy; similarly, our group proposed the use of adjusted unconventional AVS indices in order to determine the lateralization of aldosterone secretion when adrenal vein cannulation is not bilaterally selective (27).

The diagnostic performance of absolute aldosterone ratios has been analyzed only in few studies so far; Mailhot et al. (28) described the application of aldosterone index (AI), defined as the ratio of aldosterone levels between an adrenal vein and the inferior vena cava, for the assessment of selectivity; Liu et al. (29) reported a moderate accuracy of the simple AI, in sequential non-cosyntropin stimulated AVSs, for the assessment of lateralization; finally, El Ghorayeb et al. (30) demonstrated the high diagnostic accuracy of an AI <1.5 in predicting contralateral suppression and positive postoperative outcomes.

In the present study, we evaluated the performance of the aldosterone indices in predicting the selectivity of the AVS and the lateralization of aldosterone hypersecretion. The novelties of the present study were:

- The determination of AI cut-offs for the assessment of selectivity in unstimulated AVS;
- The attempt to integrate the information derived from the AI in terms of lateralization with that carried by other recognized predictors of unilateral hypersecretion, in order to create a unique clinical-/imaging-adjusted diagnostic score;
- The evaluation of the accuracy of the comparative aldosterone index (CAI), defined as the ratio of aldosterone levels in the dominant vs non-dominant adrenal vein.

## Methods

### Design and Study Population

Data of all patients that achieved a biochemical diagnosis of PA and underwent AVS for subtype differentiation, referred between 01/2009 and 12/2020 to two tertiary referral centers in northern Italy (the Division of Endocrinology, Diabetes and Metabolism of the University of Turin and the Endocrinology Unit of the University of Padua), were collected from prospective registries and analyzed retrospectively. The study followed the Standards for Reporting Diagnostic accuracy studies (STARD) (31). The protocol included patients who underwent AVS, with at least 6-12 months of follow-up after eventual adrenalectomy. Exclusion criteria were: diagnosis of aldosterone- and cortisol-co-secreting adrenal tumors and ACTH-stimulated AVS.

Approvals from local ethics committees were obtained (no. 0029680) with a central coordination by the Ethics Committee of the City of Health and Science University Hospital of Turin (ClinicalTrials.gov no. NCT04378387). The participants provided their written informed consent to participate in this study. The patients of this series were involved in a previous and different investigation (27).

### Clinical and Biochemical Investigations

Clinical and biochemical evaluations at diagnosis were collected. Office blood pressure (BP) values were measured according to guidelines (32). BP control was defined as an average office BP <140/90 mmHg. Hypokalemia was defined as serum potassium levels <3.5 mmol/L. Plasma aldosterone concentration (PAC), plasma renin activity (PRA) and PAC after saline infusion test (SIT) were determined in all patients after the replacement of interfering drugs, according to Endocrine Society guidelines (1). Furthermore, before the hormonal testing, hypokalemia was corrected, and patients were advised to maintain a normal dietary sodium intake. Patients remained in the same therapy during the entire diagnostic workup (from first-line tests to AVS). PA was diagnosed when the following criteria were met at the same time: 1) aldosterone-to-renin ratio (ARR) >300 (pg/mL)/(ng/mL/h) and 2) plasma aldosterone concentration (PAC) after saline infusion test (SIT) >100 pg/mL or ARR after captopril challenge test (CCT) >300 (pg/mL)/(ng/mL/h) or PAC reduction after CCT <30% compared to the pre-test value.

### Differentiation of PA Subtypes

In all patients with proven PA, contrast-enhanced computed tomography (CT) of the adrenal glands was performed to identify any adrenal lesions and to localize adrenal veins. Patients were considered eligible for AVS if they agreed to undergo unilateral adrenalectomy in case of demonstration of lateralized aldosterone hypersecretion. AVS was performed by two experienced radiologists between 8:00 and 11:00 AM, using sequential cannulation and without cosyntropin stimulation. No more than 15 minutes elapsed between sampling of the left and right adrenal veins. AVS was performed *via* a percutaneous femoral vein approach and the adrenal veins were cannulated using a fluoroscopy guide.

For clinical purposes, a cortisol selectivity index (CSI) >2 was considered as indicative of selective sampling. Intraprocedural cortisol measurement was routinely used in our centers to evaluate the correct placement of catheter. A LI >3 was considered to demonstrate lateralization of aldosterone production. When results of AVS were inconclusive, the repetition of AVS was offered to the patient.

All patients with lateralization of aldosterone secretion (LI >3) underwent adrenalectomy; the diagnosis of unilateral PA was confirmed after surgery by histological examination, cure or significant amelioration of hypertension, normokalemia, normal ARR and low aldosterone levels, and/or the normal suppressibility of aldosterone. Outcomes of adrenalectomy were assessed according to the PASO criteria (33).

### Aldosterone Indices

For each adrenal gland, we calculated the aldosterone index (AI, aldosterone in the adrenal vein divided by aldosterone in IVC) and the comparative aldosterone index (CAI, the ratio between aldosterone levels in the dominant and non-dominant adrenal veins, respectively). The diagnostic accuracy of AI was tested in predicting the selectivity of AVS and the final diagnosis of unilateral PA; to enhance clarity, when using the AI for these two purposes, we will refer to it as aldosterone selectivity index (ASI), and aldosterone lateralization index (ALI), respectively. The diagnostic accuracy of CAI was assessed only for the subtype diagnosis of PA. For this purpose, single adrenal glands were classified into three secretive conditions: glands with ipsilateral hypersecretion, with contralateral hypersecretion or glands within a setting of bilateral hypersecretion.

### Analytical Methods

Serum aldosterone levels (pg/mL) were measured by RIA (ALDOCTK-2, Sorin Biomedica, Saluggia, Italy). The sensitivity of the assay was 10 pg/mL; the intra- and interassay coefficient of variation ranges from 1.7% to 5.3% and from 3.4% to 7.0%, respectively. PRA (ng/mL/h) was assessed by radioimmunoassay (RENCTK, Sorin Biomedica, Saluggia, Italy). The sensitivity of the assay was 0.20 ng/mL; the intra- and interassay coefficient of variation ranges from 5.4% to 9.9% and from 7.7% to 11.5%, respectively. Serum cortisol levels (µg/L) were determined by a competitive electrochemiluminescence immunoassay automated on Cobas e601 instrument (Roche Diagnostics GmbH, Germany). Analytical sensitivity was 0.018 µg/dL (0.500 nmol/L). Intra- and inter-assay precision for serum cortisol ranged from 3.0% to 5.7% and from 2.4% to 6.2%, respectively. All other biochemical variables were assayed in plasma or serum using standard methods.

### Statistical Analysis

Baseline characteristics of all patients are summarized using median and interquartile range (IQR) for continuous non-normally distributed variables, or mean and standard deviation for normally distributed ones. Categorical variables are summarized using percent values. Shapiro-Wilk test was used to assess normality. Between-group differences in personal and clinical features were evaluated by the Student t-test or Mann-Whitney U test for continuous variables and by the chi-square test or Fisher’s exact test for categorical variables. Paired t-test or Wilcoxon signed-rank test were used for paired data comparison in patients before and after adrenalectomy. A p-value <0.05 was considered statistically significant.

Receiver-Operating Characteristic (ROC) curves were computed to evaluate the diagnostic performances of the unconventional aldosterone indices (ASI, ALI and CAI). The accuracy of ASI was evaluated in predicting selectivity according to the most diffused cut-offs for CSI (1.1, 2 and 3). The accuracy of ALI was evaluated for the reciprocal distinction between three different gland secretive conditions; more specifically, a pairwise classification analysis was performed in order to distinguish (i) glands with ipsilateral disease from those with bilateral hypersecretion and (ii) glands with contralateral disease from those with bilateral hypersecretion. Finally, the accuracy of CAI was evaluated for the distinction between unilateral and bilateral forms of the disease.

In order to refine our analysis, ALI, after a logarithmic transformation, was then included in multivariate logistic regression models, together with lesion side at imaging and the presence of hypokalemia, in order to improve its performance in the prediction of the outcome. The performances of the multivariate classifiers (the covariate-adjusted indices), obtained by the weighted combination of these predictors according to the regression coefficients of each model, were again evaluated using ROC curves. For an immediate clinical application of our results, we then calculated the thresholds of these covariate-adjusted unconventional indices in an explicit form, for all possible combinations of the other covariates (i.e., lesion side at imaging and presence/absence of hypokalemia); these thresholds were computed both according to the Youden’s criterion and after setting a pre-established specificity of 90%. Statistical analysis was performed using R 3.5.3 (R Core Team, R Foundation for Statistical Computing, Vienna, Austria, 2019).

## Results

### General Characteristics of the Study Population

**Figure 1:**
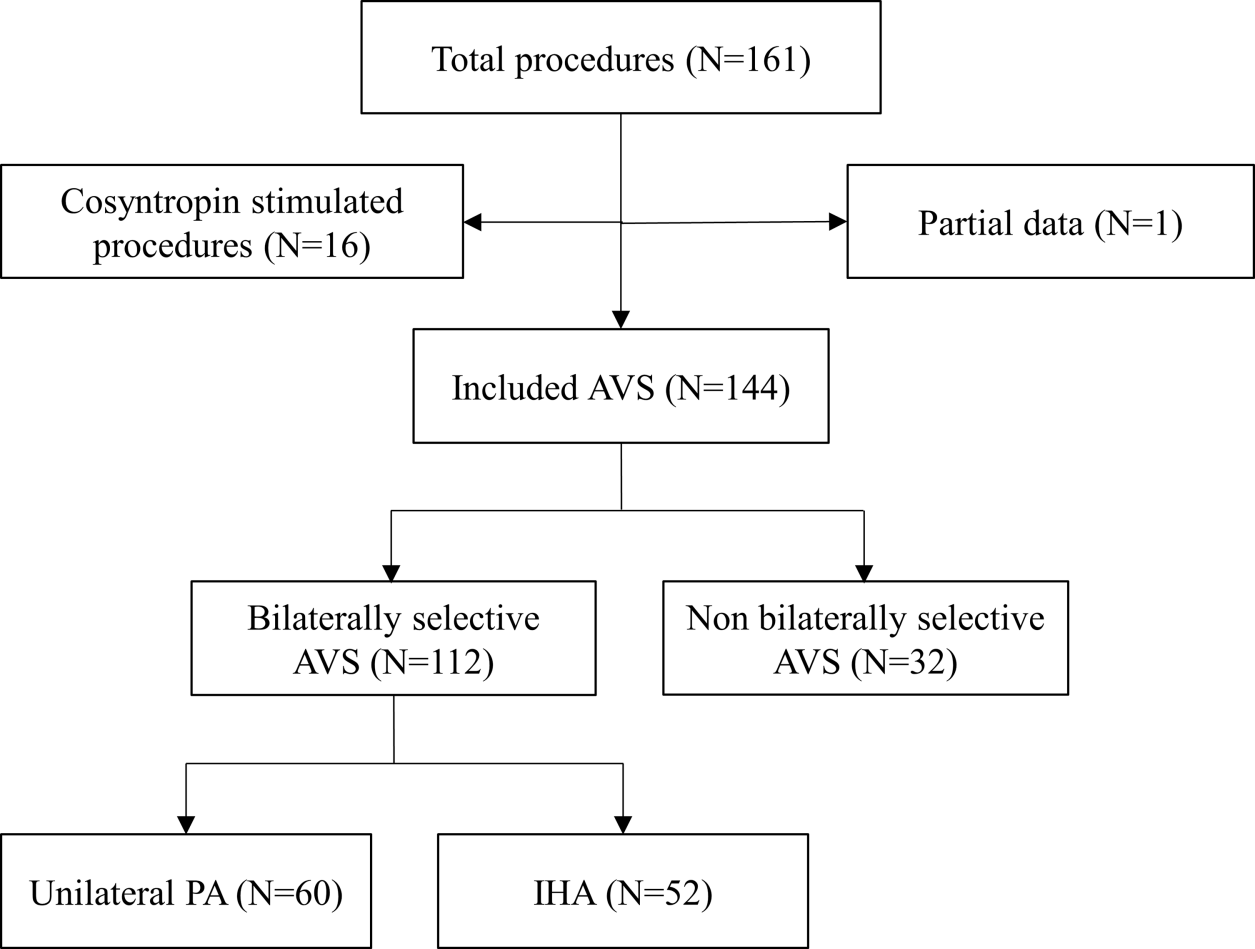
Study flow chart. AVS, adrenal vein sampling; IHA, idiopathic hyperaldosteronism; PA, primary aldosteronism.

One hundred and sixty-one AVSs were performed in our centers from 01/2009 to 12/2020. As shown in **Figure 1**, 16 cosyntropin-stimulated and 1 case with partial data were excluded from the study. Thus, 144 procedures (112 bilaterally selective and 32 non bilaterally selective) were included for the evaluation of selectivity, while only the 112 selective AVSs were considered for the assessment of lateralization. The subtype diagnosis of PA revealed 60 cases (53.6%) of lateralized aldosterone secretion (66.7% on the left and 33.3% on the right side) and 52 individuals (46.4%) with idiopathic hyperaldosteronism (IHA).

No differences between unilateral PA and IHA groups were found in gender, center of diagnosis, age at diagnosis of PA, duration of hypertension, weight, BMI, systolic blood pressure (SBP), diastolic blood pressure (DBP), number of antihypertensive drugs, sodium, PRA, lesion size and right/left CSI (**Table 1**).

**Table 1:** Clinical characteristics for all patients and for the IHA, the unilateral PA and the unclassified groups.

Variables/Parameters	Overall data (N=144)	Diagnosis			p-value
		Unclassified (N=32)	IHA (N=52)	Unilateral PA (N=60)	
Center					
* Turin*	59%	62.5%	67.3%	50%	0.161
* Padua*	41%	37.5%	32.7%	50%
Male gender	61.8%	53.1%	71.2%	58.3%	0.197
Age at diagnosis of PA (years)	49 ± 11	48 ± 12	49 ± 10	48 ± 12	0.709
Duration of AH (years)	5 (2-12)	3.5 (0-9)	8.5 (3-15)	5 (2-13)	0.346
Weight at diagnosis (Kg)	79 ± 16	80 ± 17	82 ± 17	78 ± 16	0.211
BMI (Kg/m^2^)	27.38 ± 4.35	27.66 ± 4.76	27.91 ± 4.64	26.87 ± 3.92	0.094
SBP (mmHg)	160 (140-170)	160 (148-180)	160 (143-175)	155 (140-170)	0.218
DBP (mmHg)	100 (90-109)	100 (94-105)	100 (90-110)	100 (90-101)	0.131
No. of antihypertensive drugs	2 (1-3)	2 (0-3)	2 (1-3)	2 (1-3)	0.213
Sodium (mmol/L)	142 ± 2	142 ± 3	143 ± 3	143 ± 2	0.843
Potassium (mmol/L)	3.2 ± 0.7	3.2 ± 0.8	3.6 ± 0.5	2.9 ± 0.5	<0.001
PAC (pg/mL)	380 (279-574)	374 (278-493)	348 (246-481)	445 (303-714)	0.012
PRA (ng/mL/h)	0.20 (0.10-0.60)	0.20 (0.1-0.46)	0.21 (0.10-0.68)	0.20 (0.10-0.62)	0.806
ARR [(pg/mL)/(ng/mL/h)]	1547 (694-3027)	1537 (784-3117)	1220 (606-3009)	1950 (826-3063)	0.049
PAC after SIT (pg/mL)	188 (120-271)	219 (153-285)	141 (110-203)	199 (129-309)	0.005
PAC after CCT (pg/mL)	358 (206-473)	272 (176-416)	236 (147-262)	437 (330-594)	<0.001
Lesion size (mm)	14 (10-19)	15 (10-19)	14 (10-15)	13 (10-18)	0.742
Lesion side					
* Absent/hyperplasia*	30.6%	43.8%	48.1%	8.3%	<0.001
* Left*	38.2%	25%	26.9%	55.0%
* Right*	22.2%	25%	17.3%	25.0%
* Bilateral*	9.0%	6.2%	7.7%	11.7%
Peripheral vein cortisol	127 (107-213)		156 (106-228)	156 (108-206)	0.320
Peripheral vein aldosterone	209 (180-454)	236 (162-401)	192 (148-331)	383 (228-567)	<0.001
Left adrenal vein cortisol	1068 (450-3437)	303 (193-575)	1915 (896-3705)	1952 (581-3685)	0.556
Left adrenal vein aldosterone	3406 (833-8697)	650 (353-2451)	3709 (1179-6350)	7105 (1877-18434)	0.013
Right adrenal vein cortisol	1405 (524-5536)	290 (138-895)	2945 (1594-7516)	2997 (771-5865)	0.320
Right adrenal vein aldosterone	3918 (937-12124)	777 (126-8517)	7554 (3162-14865)	2482 (937-12909)	0.047
Left CSI	8.9 (3.1-19.6)	1.8 (1.3-3.9)	13.1 (5.7-22.1)	13.1 (5.7-20.7)	0.746
Right CSI	14.6 (4.5-29.8)	1.74 (1.1-4.9)	21.5 (8.2-38.8)	17.1 (7.1-29.8)	0.355
Left AI	14.2 (2.8-39.0)	2.5 (1.1-19.0)	15.6 (6.2-32.9)	18.1 (5.2-51.8)	0.378
Right AI	14.4 (2.7-46.8)	1.8 (0.9-29.6)	29.3 (12.9-72.4)	6.9 (2.6-30.2)	<0.001

Statistical differences were calculated between the IHA and the unilateral PA groups. AH, arterial hypertension; ARR, aldosterone-to-renin ratio; BMI, body mass index; CCT, captopril challenge test; CSI, cortisol selectivity index; DBP, diastolic blood pressure; IHA, idiopathic hyperaldosteronism; PA, primary aldosteronism; PAC, plasma aldosterone concentration; PRA, plasma renin activity; SBP, systolic blood pressure; SIT, saline infusion test.

As expected, when compared with IHA patients, unilateral PA individuals had lower levels of potassium (2.9 ± 0.5 vs 3.6 ± 0.5 mmol/L; p<0.001), higher values of PAC (median 445, IQR 303-714 vs 348, 246-481 pg/mL; p=0.012), ARR [1950, 826-3063 vs 1220, 606-3009 (pg/mL)/(ng/mL/h); p=0.049], PAC after SIT (199, 129-309 vs 141, 110-203 pg/mL; p=0.005), PAC after CCT (437, 330-594 vs 236, 147-262; p<0.001), peripheral vein (383, 228-567 vs 192, 148-331 pg/mL; p<0.001) and left adrenal vein (7105, 1877-18434 vs 3709, 1179-6350 pg/mL; p=0.013) aldosterone levels. Unilateral PA group showed lower levels of right adrenal vein aldosterone (2482, 937-12909 vs 7554, 3162-14865 pg/mL; p=0.047), lower rate of bilateral adrenal hyperplasia/normal adrenal glands at CT (8.3 vs 48.1%; p<0.001) and higher rate of left (55.0 vs 26.9%; p<0.001) and right adrenal nodules (25.0 vs 17.3%; p<0.001), compared to IHA patients (**Table 1**).

### Post-Surgery Outcomes

Histological examination discovered 51 cases of APA, 6 adrenal hyperplasia and 3 micronodular hyperplasia. The re-evaluation at 6-12 months after adrenalectomy, according to PASO criteria (33), demonstrated the positive outcome of surgical treatment on BP, sodium, potassium, number of antihypertensive drugs, ARR, PAC and PAC after SIT, without significant effect on weight and BMI (**Table S1**). Complete biochemical success was achieved in 96.7% and partial in 3.3% of patients. Complete clinical success was reached in 41.7% and partial in 58.3% of patients. Outcomes were not different between patients with APA, adrenal hyperplasia and micronodular hyperplasia, but this observation is limited by the small number of patients with other than APA forms of unilateral PA. All patients that achieved complete clinical success were amongst those who also achieved complete biochemical success (data not shown).

### Accuracy of Aldosterone Indices

#### Aldosterone Selectivity Index (ASI)

**Figure 2:**
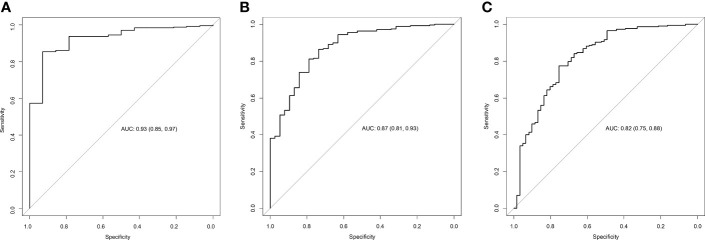
Accuracy of the AI for predicting selectivity of AVS. ROC curves describing the accuracy of AI for predicting selectivity using as reference a cortisol selectivity cut-off of 1.1 **(A)**, 2 **(B)** and 3 **(C)**. AUC, area under the curve; AVS, adrenal vein sampling; AI, aldosterone index.

The accuracy of ASI was assessed for determining the correct cannulation of adrenal veins, using as reference standards the most diffused cut-offs for CSI (1.1, 2 and 3). As shown in **Figure 2** and **Table 2**, ASI demonstrated a high accuracy in predicting the selectivity using CSI cut-off of 1.1 as the reference standard (AUC 0.93, 95% CI 0.85-0.97, cut-off >1.67, Se 85%, Sp 93%, +LR 5, -LR 0.16; **Figure 2A** and **Table 2**), and a moderate accuracy if compared with CSI of 2 (AUC 0.87, 95% CI 0.81-0.93, cut-off >2.87, Se 81%, Sp 79%, +LR 3.9, -LR 0.24; **Figure 2B** and **Table 2**) and 3 (AUC 0.82, 95% CI 0.75-0.88, cut-off >4.83, Se 78%, Sp 75%, +LR 3.1, -LR 0.29; **Figure 2C** and **Table 2**).

**Table 2:** Accuracy of aldosterone indices.

	Cut-off	AUC (95% CI)	Se	Sp	+LR	-LR
**CSI cut-offs**	**ASI**					
*1.1*	>1.67	0.93 (0.85-0.97)	85	93	5.00	0.16
*2*	>2.87	0.87 (0.80-0.93)	81	79	3.50	0.24
*3*	>4.83	0.82 (0.75-0.88)	78	75	3.10	0.29
**Predicted side of hypersecretion**	**Simple ALI**					
*Ipsilateral^#^*	>26.63	0.71 (0.62-0.78)	77	61	1.97	0.38
*Contralateral^#^*	<9.20	0.84 (0.78-0.90)	80	76	3.33	0.26
**Predicted subtype**	**CAI**					
*Unilateral PA*	>5.02	0.92 (0.87-0.96)	82	92	10.3	0.20

Cut-offs, AUC, sensitivity, specificity, +LR and -LR of ASI, simple ALI and CAI, according to the Youden’s criterion. ALI, aldosterone lateralization index; AUC, area under the curve; ASI, aldosterone selectivity index; CAI, comparative aldosterone index; CSI, cortisol selectivity index; -LR, negative likelihood ratio; +LR, positive likelihood ratio; PA, primary aldosteronism; Se, sensitivity; Sp, specificity.

^#^Versus bilateral hypersecretion.

#### Aldosterone Lateralization Index (ALI)

**Figure 3:**
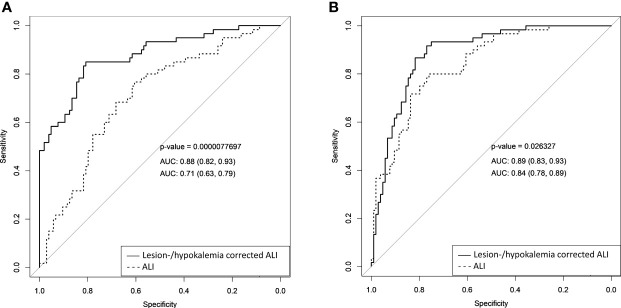
Comparison of ROC curves of simple and corrected AI for predicting lateralization of aldosterone hypersecretion. Comparison of ROC curves of simple and lesion-/hypokalemia-corrected AI in the prediction of ipsilateral **(A)** and contralateral **(B)** aldosterone hypersecretion. AUC, area under the curve; AI, aldosterone index.

The diagnostic accuracy of ALI was assessed to distinguish 1) glands with ipsilateral disease from those with bilateral hypersecretion and 2) glands with contralateral disease from those with bilateral hypersecretion. ALI showed a moderate accuracy in predicting ipsilateral disease (AUC 0.71, 95% CI 0.63-0.79, cut-off >26.63, Se 77%, Sp 61%, +LR 1.97, -LR 0.38) and contralateral aldosterone hypersecretion (AUC 0.84, 95% CI 0.78-0.89, cut-off <9.20, Se 80%, Sp 76%, +LR 3.33, -LR 0.26) (**Figure 3** and **Table 2**).

#### Covariate-Adjusted ALI

Multivariate logistic regression models, considering as covariates lesion side at CT and hypokalemia, were used to create lesion- and hypokalemia-corrected ALI.

Lesion side at imaging was initially considered as a three-level (ipsilateral lesion, contralateral lesion, bilateral/no lesions) unordered categorical variable; however, in all models predicting ipsilateral disease, no statistically significant differences were found between the “contralateral lesion” and the “bilateral/no lesions” categories; similarly in all models predicting contralateral disease, no statistically significant differences were found between the “ipsilateral lesion” and the “bilateral/no lesions” categories. Therefore, all logistic regression models were simplified by merging the lesion categories that did not significantly differed, thus reducing lesion side at imaging to a binary variable. Complete data about the final logistic regression models are available in the supplementary material (**Tables S2, S3**).

The ROC curves, evaluating the performances of the lesion-/hypokalemia-corrected ALI, revealed a significant improvement in the accuracy of predicting ipsilateral (AUC 0.88, 95% CI 0.82-0.93, Se 85%, Sp 81%, +LR 4.5, -LR 0.19; **Figure 3A**) and contralateral (AUC 0.89, 95% CI 0.83-0.93, Se 92%, Sp 77%, +LR 4.0, -LR 0.10; **Figure 3B**) disease, in comparison with simple ALI. The improvement of covariate-adjusted unconventional indices was statistically significant in all models (**Figure 3**). In addition to the cut-offs identified by Youden’s criterion, those corresponding to a pre-established specificity of 90% were also considered (**Table S4**). For an immediate clinical application of our results, we then calculated the thresholds of these covariate-adjusted unconventional indices in an explicit form, for all possible combinations of the other covariates (i.e., lesion side at imaging and presence/absence of hypokalemia); these thresholds were computed both according to the Youden’s criterion (**Table S5**) and after setting a pre-established specificity of 90% (**Table 3**).

**Table 3:** Thresholds of lesion side- and potassium-corrected ALI for the diagnosis of ipsilateral/contralateral aldosterone hypersecretion, setting a specificity of 90%.

Index	Predicted side of hypersecretion	Normokalemia			Hypokalemia		
		Ipsilateral lesion	Bilateral/no lesions	Contralateral lesion	Ipsilateral lesion	Bilateral/no lesions	Contralateral lesion
**ALI**	**Ipsilateral^#^**	>157.51	>1063.91	>1063.91	>13.08	>88.35	>88.35
	**Contralateral^#^**	<0.53	<0.53	<3.34	<1.34	<1.34	<8.36

ALI, aldosterone lateralization index.

^#^Versus bilateral hypersecretion.

#### Comparative Aldosterone Index (CAI)

**Figure 4:**
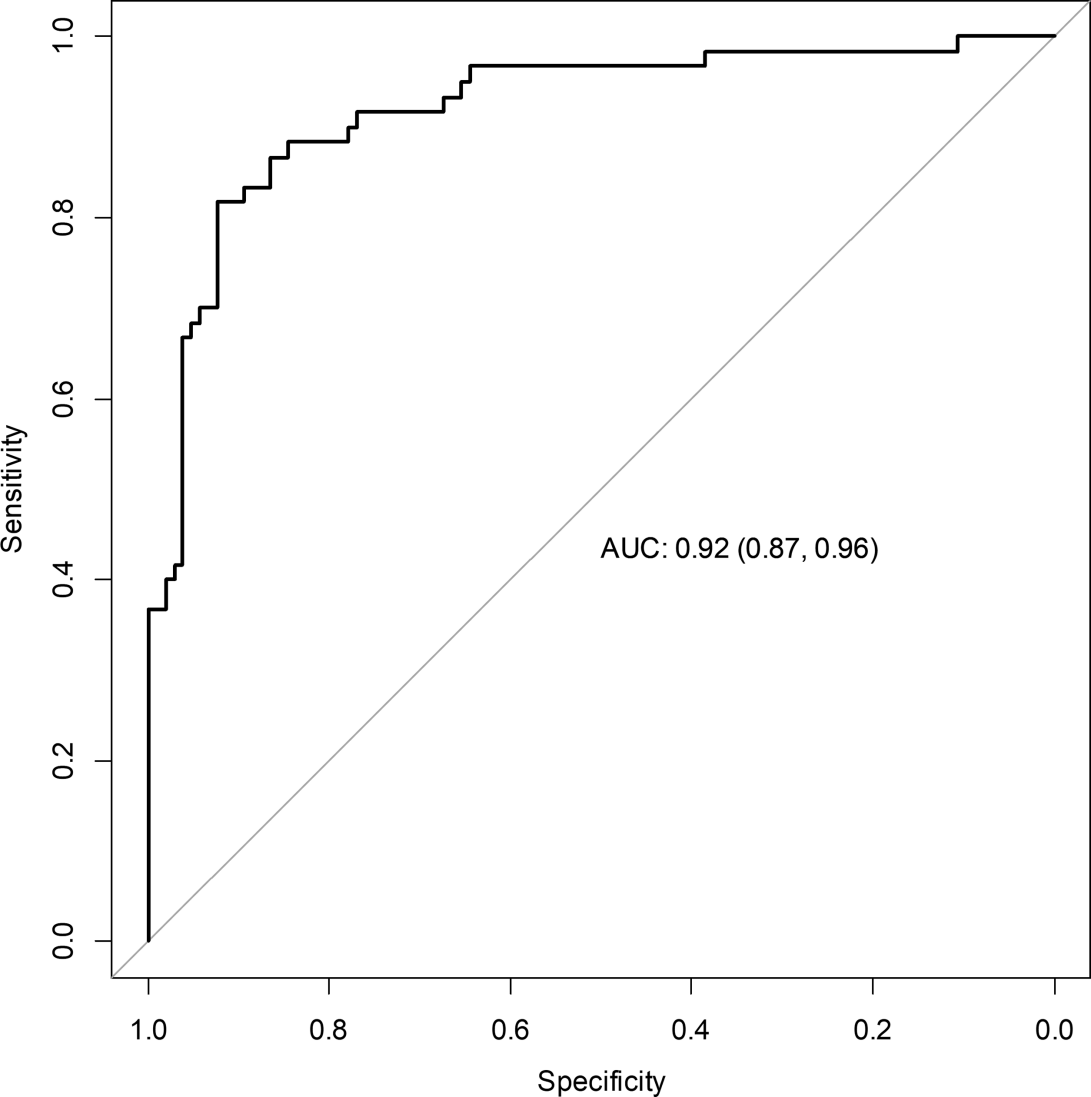
Accuracy of CAI for predicting lateralization of aldosterone hypersecretion. ROC curve describing the accuracy of CAI for predicting the lateralization of aldosterone hypersecretion. AUC, area under the curve; CAI, comparative aldosterone index.

CAI revealed a high accuracy in predicting the subtype diagnosis (AUC 0.92, 95% CI 0.87-0.96, Se 82%, Sp 92%, +LR 10.3, -LR 0.20) with a cut-off >5.02 for unilateral aldosterone hypersecretion (**Figure 4** and **Table 2**).

## Discussion

The present study seemed to endorse the application of the AI in predicting selectivity and lateralization of AVS in patients with PA. We studied the AI as a selectivity index (ASI) and as lateralization index (ALI), which both provided a satisfactory accuracy, particularly for ALI, that became even more accurate after the correction for hypokalemia and lesion side. Moreover, we introduced the CAI, a straightforward aldosterone ratio between the adrenal glands, that showed a high accuracy in the assessment of lateralization of aldosterone hypersecretion.

International guidelines and expert consensus (1, 2, 34–36) recognize AVS as the gold standard for the diagnosis of PA subtypes, however, AVS is used only in few centers worldwide, due to technical difficulties in the interventional radiology approach. Without a bilaterally selective procedure, the LI, which is the most widely accepted index in the literature, is not applicable to guide the diagnosis of lateralization of aldosterone hypersecretion and the subsequent opportunity of surgical treatment. Therefore, the introduction of unconventional indices and statistical models (25–27, 37, 38) could help in determining the lateralization of aldosterone secretion in case of suboptimal AVS. Our group recently demonstrated the high accuracy of corrected unconventional indices in the prediction of unilateral disease in a large series of patients with PA subtyped with AVS (27). In the present study, we focused on the reliability of aldosterone indices for both the selectivity of the sampling and the lateralization of aldosterone hypersecretion. In the literature, Mailhot et al. (28) described the utility of ASI and CSI (alone or combined) during unstimulated AVS in predicting selectivity, using the same pre-established cut-offs used for the CSI (1.1, 2 and 3) and comparing their accuracy with a composite standard (CSI and/or ALI >5 during ACTH-stimulated AVS). In our study, we derived 3 new cut-offs for ASI: 1.67 compared to a CSI of 1.1, 2.87 compared to a CSI of 2, and 4.83 compared to a CSI of 3. It is clear how the retrieved aldosterone cut-offs are quite different in comparison to the cortisol ones.

Regarding the role of AI in determining lateralization of aldosterone secretion, Liu et al. (29) tried to prove the utility of AI in determining the dominant adrenal gland. While El Ghorayeb et al. (30) demonstrated the high diagnostic accuracy of AI <1.5 in predicting contralateral suppression and positive postoperative outcomes.

In the present study, the performance of simple ALI was higher in the prediction of contralateral (rather than ipsilateral) aldosterone hypersecretion, but the correction for lesion side and hypokalemia provided a significant improvement in the diagnostic accuracy of this index for the prediction of both ipsi- and contralateral lateralization. It has to be highlighted that we should be sure of a correct cannulation of the adrenal vein when we use the ALI for predicting contralateral hypersecretion; in fact, a low ALI value could be present also in the case of an inadequate selectivity of the AVS. To avoid false positive results that may lead to the surgical removal of an adrenal gland in the context of IHA, we also decided to provide cut-offs with optimized specificity. Moreover, we derived the thresholds of covariate-adjusted ALI in an explicit form, in order to give an immediate clinical applicability to the study.

In this study, we also introduced the CAI, a straightforward ratio of aldosterone in the two adrenal veins, which demonstrated a high accuracy in predicting lateralization of aldosterone hypersecretion. However, being a ratio between aldosterone values in the two adrenal veins, the application of this index needs a bilaterally selective AVS.

The strengths of our study are the very low rate of false positive diagnosis of PA, due to the diagnosis of florid cases of PA and to the diagnostic experience of our centers, which confers homogeneity to the studied population; the longstanding expertise in performing AVS of dedicated radiologists working in our centers; the presence of rigidly defined AVS criteria to assess lateralization (using a LI >3) and to guide the surgical indication; the presence of subsequent follow-up and the application of widely accepted criteria for the evaluation of outcomes in surgical treated patients, obtaining outcomes that proved to be consistent with literature (33).

It should be noted that in the present study the reliability of unconventional indices was derived only in comparison with the IHA group, with a potential underestimation of the real accuracy of these indices. Despite this choice, we showed that the reached reliability is highly satisfactory.

The present study has some limitation. First, its retrospective design, even if data were collected from prospective registries. Second, the potential for selection bias due to the tertiary nature of our centers. Third, our results apply to unstimulated AVS with a correct selectivity in at least one adrenal vein. Moreover, data should be validated in an external cohort before the introduction in clinical practice.

### Contribution of the Study to the Field and Conclusions

Our study suggests the possibility of accurately determining selectivity (ASI) and lateralization of the aldosterone secretion (ALI and CAI) without using cortisol-corrected indices and, exclusively for ALI, even if AVS is not bilaterally selective. We know that the lack of accepted standards for the performance of selective AVS and the interpretation of its results contributes to the hesitancy towards the adoption of the unconventional indices in the case of non-conclusive AVS data. In this way, however, many PA patients are denied curative surgery or undergo adrenalectomy without the evidence of lateralization, which may result in the removal of an adrenal gland in a patient with IHA. PA is a frequent cause of organ damage among patients with arterial hypertension, and aldosterone has a key role in the pathogenesis of cardiovascular disease. Considering the low probability of spontaneous remission of florid PA (39), the possibility to cure PA with adrenalectomy, the improvement of organ damage (40–42) and the reduced incidence of cardiometabolic complications with the resolution of hyperaldosteronism (43–45), the research of alternative/unconventional indices for the interpretation of AVS, even in case of interfering factors or suboptimal sampling, is of crucial importance.

Given our results, we suggest the use of the proposed unconventional aldosterone indices (ASI, ALI, CAI) to further confirm the selectivity and the lateralization of AVS, in addition to conventional SI and LI. Moreover, they could help in the interpretation of unilaterally or bilaterally selective AVS and in the selection of patients for adrenalectomy in all cases in which the measurement of cortisol levels could be unreliable, such as in patients taking chronic steroid treatment, and in the case of pre-analytical errors that denied a complete hormonal assessment. These indices may be also considered in patients with aldosterone/cortisol cosecretion or the so-called Conn-shing syndrome, even if patients with cosecretion have been excluded from this study. Further *ad hoc* studies are necessary for a definitive validation of this last indication.

## Data Availability Statement

The raw data supporting the conclusions of this article will be made available by the authors, without undue reservation.

## Ethics Statement

Approvals from local ethics committees were obtained (no. 0029680) with a central coordination by the Ethics Committee of the City of Health and Science University Hospital of Turin (ClinicalTrials.gov no. NCT04378387). The patients/participants provided their written informed consent to participate in this study.

## Author Contributions

Conceptualization: MP, FB, FC, and MM. Methodology: MP and FB. Validation: CL, MB, MC, and GV. Resources: MB, DR, and GG. Data Curation: FB, FC, CL, and GV. Writing – Original Draft Preparation: MP and FB. Writing – Review & Editing: DR, GG, CS, EG, and MM. Supervision: CS, EG, and MM. All authors contributed to the article and approved the submitted version.

## Funding

The study was supported by grants from the University of Turin and the University of Padua. The funding sources had no role in the study design, data collection and analysis, manuscript preparation, or decision to submit the paper for publication.

## Conflict of Interest

The authors declare that the research was conducted in the absence of any commercial or financial relationships that could be construed as a potential conflict of interest.

## Publisher’s Note

All claims expressed in this article are solely those of the authors and do not necessarily represent those of their affiliated organizations, or those of the publisher, the editors and the reviewers. Any product that may be evaluated in this article, or claim that may be made by its manufacturer, is not guaranteed or endorsed by the publisher.
