# Supplementary material for: The Accuracy of Simple and Adjusted Aldosterone Indices for Assessing Selectivity and Lateralization of Adrenal Vein Sampling in the Diagnosis of Primary Aldosteronism Subtypes
Source: Front Endocrinol (Lausanne). 2022 Feb 16;13:801529. doi: 10.3389/fendo.2022.801529 (PMC8888437; doi:10.3389/fendo.2022.801529)
Supplement: Supplementary file 1 [file DataSheet_1.docx]

### **Supplement data file**

**Tables**

| **Variables/Parameters** | **At diagnosis** | **After surgery** | **p-value** |
| --- | --- | --- | --- |
| Weight (Kg) | 78±16 | 77±13 | 0.693 |
| BMI (Kg/m^2^) | 27.38±4.35 | 27.14±3.92 | 0.235 |
| SBP (mmHg) | 155 (140-170) | 130 (120-134) | <0.001 |
| DBP (mmHg) | 100 (90-101) | 80 (80-85) | <0.001 |
| No. of antihypertensive drugs | 2 (1-3) | 1 (0-2) | 0.010 |
| Sodium (mmol/L) | 143±2 | 140±3 | <0.001 |
| Potassium (mmol/L) | 2.9±0.5 | 4.2±0.5 | <0.001 |
| PAC (pg/mL) | 445 (303-714) | 112 (80-184) | <0.001 |
| PRA (ng/mL/h) | 0.20 (0.10-0.62) | 2.19 (0.49-3.84) | <0.001 |
| ARR [(pg/mL)/(ng/mL/h)] | 1547 (694-3028) | 113 (39-402) | <0.001 |
| PAC after SIT (pg/mL) | 199 (129-309) | 60 (17-85) | <0.001 |

**Table S1.** Clinical data of patients with unilateral PA before and after adrenalectomy.

Abbreviations: ARR, aldosterone-to-renin ratio; BMI, body mass index; DBP, diastolic blood pressure; PA, primary aldosteronism; PAC, plasma aldosterone concentration; PRA, plasma renin activity; SBP, systolic blood pressure; SIT, saline infusion test.

| **Covariate** | **Coefficient at**  **logistic regression** | **OR** | **95% CI for OR** | **p-value** |
| --- | --- | --- | --- | --- |
| Log_10_ AI | 2.23 | 9.30 | 3.57-24.19 | <0.001 |
| Ipsilateral lesion  at imaging | 1.85 | 6.38 | 2.71-15.02 | <0.001 |
| Hypokalemia | 2.41 | 11.09 | 4.10-30.00 | <0.001 |

**Table S2.** Multivariate logistic regression model for the distinction of glands with ipsilateral disease from those with bilateral hypersecretion using log_10_ AI, lesion side at imaging and hypokalemia. The reported coefficients at logistic regression are those describing the relationship between the predictor variables and the log-odds of the outcome, according to the well-known formula *log_e_ p/(1-p) = β_0_ + β_1_x_1_ + β_2_x_2_ + β_3_x_3_*.

Abbreviations: CI, confidence interval; AI, aldosterone index; OR, odds ratio.

| **Covariate** | **Coefficient at**  **logistic regression** | **OR** | **95% CI for OR** | **p-value** |
| --- | --- | --- | --- | --- |
| Log_10_ AI | -2.06 | 0.13 | 0.05-0.30 | <0.001 |
| Contralateral lesion  at imaging | 1.64 | 5.16 | 2.21-12.02 | <0.001 |
| Hypokalemia | 0.82 | 2.26 | 0.87-5.90 | 0.096 |

**Table S3.** Multivariate logistic regression model for the distinction of glands with contralateral disease from those with bilateral hypersecretion using log_10_ AI, lesion side at imaging and hypokalemia. The reported coefficients at logistic regression are those describing the relationship between the predictor variables and the log-odds of the outcome, according to the well-known formula *log_e_ p/(1-p) = β_0_ + β_1_x_1_ + β_2_x_2_ + β_3_x_3_*.

Abbreviations: CI, confidence interval; AI, aldosterone index; OR, odds ratio.

| **Index** | **Predicted side of hypersecretion** | **Model equation** | **Equation cut-offs**  **(Youden’s criterion)** | | | **Equation cut-offs**  **(optimized specificity)** | | |
| --- | --- | --- | --- | --- | --- | --- | --- | --- |
|  |  |  | **Cut-off** | **Se** | **Sp** | **Cut-off** | **Se** | **Sp** |
| AI | Ipsilateral | 2.23·log_10_AI + 1.85·IL + 2.41·HK | > 5.54 | 85% | 81% | > 6.75 | 60% | 90% |
|  | Contralateral | 2.06·log_10_AI – 1.64·CL – 0.82·HK | < 1.05 | 92% | 77% | < -0.56 | 62% | 90% |

**Table S4.** Cut-offs, sensitivity, and specificity of the model equations of lesion- and hypokalemia-corrected unconventional indices, according to the Youden’s criterion and by setting specificity at 90%. In the model equations, IL and CL should be considered as binary variables equal to 1 if an ipsilateral/contralateral lesion is present at imaging and equal to 0 if not. The meaning of HK should be interpreted similarly. Abbreviations: CL, contralateral lesion; HK, hypokalemia; IL, ipsilateral lesion; AI, aldosterone index; Se, sensitivity; Sp, specificity.

| **Index** | **Predicted side of hypersecretion** | **Normokalemia** | | | **Hypokalemia** | | |
| --- | --- | --- | --- | --- | --- | --- | --- |
|  |  | **Ipsilateral lesion** | **Bilateral/no lesions** | **Contralateral lesion** | **Ipsilateral lesion** | **Bilateral/no lesions** | **Contralateral lesion** |
| **AI** | Ipsilateral | >45.16 | >305.00 | >305.00 | >3.75 | >25.33 | >25.33 |
|  | Contralateral | <3.23 | <3.23 | <20.22 | <8.09 | <8.09 | <50.57 |

**Table S5.** Thresholds of lesion side- and potassium- corrected AI for the diagnosis of ipsilateral/contralateral aldosterone hypersecretion, according to the Youden’s criterion. Abbreviations: AI, aldosterone index.
